# Mr. Machell's Cases of Scarlatina Anginosa

**Published:** 1811-12

**Authors:** Thomas Machell

**Affiliations:** Wolsingham, Durham


*To the Editors of the Medical and Physical Journal.*

Gentlemen,

If the Cases inclosed merit a place in your valuable Journal, I will thank you to insert them, and will do myself the pleasure of giving any further information in my power, should it be required.

I am, Gentlemen,
Your most obedient Servant,
THOMAS MACHELL.

*Wolsingham, Durham,* 28*th Sept.* 1811.

From the fatal nature of the Scarlatina Anginosa, the little success of the remedies employed, and the very short period in which an opportunity can be had for the practitioner to do more than become a mere spectator to the dreadful shades which this disease so frequently assumes; we may reasonably conclude that our present state of medical knowledge is deficient: any thing, therefore, that may rouze our attention, or that may tend to put us upon our guard, must be acceptable to the feeling part of the medical profession. The chief object is not to omit the first, and in my opinion, the only opportunity of giving assistance. In many cases, if deferred until to-morrow, it never can be accomplished; and the object of our regard is hurried, in the space of a few days, a miserable sight, from his friends to perpetual silence.

Cases where success has been obtained in the application of any remedy, or which may assist in attracting the attention of practitioners to a disease which requires every exertion to subdue it in its first stage, and which may prevent that alarm we so often experience from delay, will, it is presumed, be always acceptable to your readers. In your valuable Journal for February, 1811, there are several well detailed cases of the scarlatina anginosa, treated by Dr. R. Hamilton and his nephew. I feel much gratification in saying, that I have followed their mode of treatment pretty closely in the following cases, which I have ventured to lay before the public, sincerely wishing that they may tend to establish a better mode of treating the scarlatina anginosa.

CASE I.

The family of Mr. Cumming of Cornsey, near Durham, in May, 1811, was visited by the scarlatina anginosa in a very inveterate form. A fine boy, ten years of age, was taken ill on the night of the 18th, immediately on his return from school, and died on the 21st. The second, a girl aged five, begun on the 23d and died on the 25th. The same night, a girl twelve years of age, was seized with the fever, and died on the 27th. On the first of June, a most amiable young woman of the same family, fifteen years of age, was the next victim of this formidable disease. I was sent for on the fourth of June, which was the first time of my attending the family, and saw her at six in the evening; but was only in time to witness the awful scene; for in a few hours death terminated her sufferings.

The following day a young man, brother to the above, seventeen years of age, began with the fever. I observed, the night before, he was dull, heavy and weary, and mentioned my opinion to his friends, requesting to be informed immediately if he grew worse. My apprehensions proved true; for after passing a restless night, he was attacked about six in the morning with severe pain in his head, sickness, vomiting and purging; followed almost immediately by delirium. As I resided at the distance of seven miles, it was ten o'clock in the forenoon before I saw him, during which short time all the above symptoms had become extremely aggravated. His whole body was covered with a scarlet rash; his eyes looked wild and fiery; stools were frequent, dark, fœtid, and watery; pulse quick and strong, 130 strokes in a minute; throat sore; tongue and fauces covered with aphthæ; thirst great, and liquids were swallowed with uncommon eagerness.

Mr.

Mr. C. whose paternal affection had induced him to attend closely to his family during their illness, says that this patient, for the time, was far worse then any of the rest; and that the treatment pursued by two respectable medical gentlemen that were employed by his family previous to this case, was chiefly by bark, wine, and emetics: however, from the formidable shape of the disease in this family; the frequent disappointment in the above remedies; and the fortunate opportunity I just before had in meeting with that Number of your Journal, which contains Mr. Hamilton's treatment of the scarlatina anginosa by venesection, &c. I was strongly induced to abandon, as much as possible, all the other modes of treating this disease; for if a prognosis of the present case might be drawn from the past, the time, I concluded, would be very short indeed, in which something very effectual ought to be done. Into the room, which had previously been kept very close, a free access of air was admitted by the window; the fire was extinguished; the bedding changed: this I thought prudent to do, as all the family that suffered had died in one bed. The corpses were immediately interred, and every necessary precaution taken to prevent the spreading of the contagion. The heat of this patient's body was 109° of Fahrenheit; pulse 130, and strong. I took from his arm twelve ounces of blood, and in a few minutes employed the cold water: he was well sponged for the space of three minutes, and during its use seemed quite easy, and would frequently exclaim, "Wash me well!" The heat was readily reduced to 104°, his pulse to 115; delirium had subsided, and his head was quite free from pain. Half an hour after this, the purging and vomiting abated, and I gave the saline mixture. His throat was well rubbed with the spiritus ammoniæ, which gave immediate relief to it. His mouth was well gargled with equal parts of acetum distillatum and water, a tea-spoonful of which was frequently swallowed: he complained of its being sharp; but it had the good effect of producing a copious expectoration of phlegm. In three quarters of an hour from the time he was sponged, the heat of his body had increased, the purging and sickness returned, and he talked incoherently. The same happy effect was again obtained by sponging; and in the space of six hours it was repeated eight times, or as often as necessity pointed out; for he would frequently request to be washed, and when long delayed the heat of his body increased, and every particular symptom got worse, which invariably and almost immediately gave way on applying the water. Other professional engagements obliged me to leave him at eight in the evening, instructing his father in the use of the thermometer, and to know know when the body was in a proper state for sponging. I was sent for again at twelve o'clock, midnight; he was again delirious in a very great degree, and it was difficult to confine him in bed; the heat of his body was 108, pulse 130, rather strong; the vomiting and purging had returned, his face was tinged, his lips, tongue, and teeth, of a darkish brown colour, and his eyes were very red; he had not been sponged for the last three hours, the purging returned and they had left it off; as the skin was still hot and dry, I immediately applied the water as before, with the same agreeable effect; the whole of the above symptoms readily abated, except the purging, for which I gave four drops of the tincture of opium in an ounce and a half of the saline mixture, and ordered it to be repeated every four hours. The next morning I saw him again and the purging had entirely ceased; he had been sponged thrice, and during the night had several short intervals of sleep.

On the 7th the sponging was employed twice, the last of which was followed by a gentle diaphoresis, which before never could be obtained. Saline mixture continued without the tincture of opium. Sponging omitted.

8th. Pulse 100, and soft, no delirium, thirst abated, the aphthæ covering the inside of the mouth, tongue, and fauces casting off in sloughs, tongue frequently bleeds from the whole surface, complains of great soreness of the mouth and griping pain in his bowels. R. Ol. olivar ℥iv. tinct. opii, gtt. xl. ft. enema.

9th. Pain in the bowels much relieved from yesterday's enema; had two stools this morning; mouth not so sore; some sleep in the night; thirst moderate. Saline mixture continued.

10th. Rested a little during the night; appetite better; pulse 96. Pain in the bowels quite gone, the scarf skin peeling off.

12th. Mending in every particular symptom; thirst quiet; tongue moist; sleep natural; and finds himself stronger.

14th. General health much improved, and since the last report has been quite easy, except a slight pain in the umbilical region, which he has felt since last night. R. Ol. ricini ℥ss. 2nda quaque hora ad operationem usque.

15. Since yesterday has had three stools; he took twice of the castor oil, and its operative effect is perfectly easy, and to all appearance is improving very fast.

18th. Great improvement in every particular, only he complains of slight pains in his joints on endeavouring to walk, yet is entirely free from œdematous swelling. Appetite still good; good; bowels soluble, and in every respect is fast approaching to his former health.

### CASE II.

While attending Mr. Cumming's family in the case just related, a boy, eight years of age, of a weak constitution, brother to the above, on the 10th of the same month was suddenly seized with nausea, shivering, pain in his head, no appetite, and frequent purging and vomiting. I saw him about five hours after the commencement of these symptoms, during which time the nausea, vomiting and purging had continued to alternate in a very alarming degree; the pain of his head was quite severe; his face, breast, and arms, were slightly tinged with red; neck painful and stiff, throat beginning to be sore, and the heat of his body varied from 104 to 107 degrees. I employed the same powerful means as those already related in his brother's case, and the result was still more favourable, for by the 18th he had recovered so far as to be able to walk, and appears at this time, as I had marked in my notes, gaining strength faster than his brother. This family, at the commencement of their illness, were eight in number; an infant nine weeks, and a girl two years and an half old, escaped the infection; two recovered, and four died.

### CASE III.

Mr. Borrow, of Hall-hill, in the same neighbourhood, had a family of seven children, all of which, except a boy two years old, took the scarlatina anginosa; the first that begun was a girl six years of age, I first saw her on the evening of the 19th of May, 1811, she had been ill six days, and was then lying upon her back in bed, in a comatose state; there was a fœtid discharge from her mouth; her lips, tongue, and teeth, were covered with very dark aphthæ; she breathed with great difficulty, the effluvia of which was intolerable; she had not been able to swallow any nourishment since the morning, and in this state she continued until the following morning, when the lamentable scene ended in death.

### CASE IV.

Mr. Borrow's eldest son, aged 23, of a full habit and strong constitution, begun, on the night of the sixth of June, with violent fits of shivering, alternated by heat, much pain of the head, sickness, and vomiting. I saw him early the following morning, the sickness and vomiting were become very distressing, had a violent pain in his head, strong and and quick pulse, inflamed tonsils, sore throat, and a wide spreading flush almost over his whole body, the heat of which was 108, his face was tumid, and eyes very red; from his arm I took twelve ounces of blood, and in less than an hour the violence of the whole symptoms was very much abated, so much so, that I determined on trying a little further before I applied the cold water, in any shape; I gave saline mixture and allowed a little cold water, which he drank occasionally; his throat was well rubbed with the spiritus ammoniæ. As he was very plethoric, and benefit had already been experienced from the first blooding, I took another pound of blood from the same orifice, which produced a slight degree of syncope, and which was immediately followed by a copious diaphoresis; at the end of two hours from the last bleeding there was still a more visible abatement of every particular bad symptom, and he felt very drowsy. I visited him the next morning, he had slept a great part of the night and appeared in every respect in a fair way of recovery; I ordered his bowels to be kept moderately open with small doses of calomel; he recovered in the space of five days.

All Mr. Borrow's family had the scarlatina anginosa in a somewhat similar form; and all, with an exception to the first that took it, ultimately recovered under the same mode of treatment: to save prolixity I have made choice of his eldest son's case, as being the most decided in serving to illustrate the beneficial effects of venesection.

### CASE V.

Mr. W. Dodds, 26 years of age, was the next bad case that I attended; he had felt himself excessively weary, and frequently chilly, with fugitive pains for a day or two previous to the attack. When I first saw him the skin over his whole body was a bright red, his face swollen, throat, tongue, and palate, covered with aphthæ, violent pain in his head, sickness and vomiting, his pulse full and rapid, and the adnata of his eyes had a very red suffusion. I extracted from his arm fourteen ounces of blood,* this was the first day of the rash appearing, and in the space of three hours its good effects were very decisive, for, excepting the saline mixture and a purge occasionally, there was not any necessity for more medicine. On the following morning I saw him again, and his arm some time in the night had by accident bled from

* I confess my boldness in this practice, particularly when I have the good fortune to be sent for soon after the commencement of the fever the the same orifice, probably to the quantity of another six ounces;* he had had some sleep, was quite clear of pain in his head, and excepting general weakness, he was free from any other complaint.

### CASE VI.

——— Tayler, aged 23, servant to Mr. Dodds, of Adelpha, was very stout and robust, he had the scarlatina anginosa. I was sent for on the 12th of June, he had been ill two days, and was then rather delirious, had severe pain in his head, strong quick pulse, difficult deglutition, sore throat, no appetite, and much thirst; his body was all over very red. I took a pound and a half of blood from his arm, and ordered the saline mixture; his throat was well rubbed with the spiritus ammoniæ, and in the space of a few hours the relief he had experienced prevented the necessity of employing affusion or sponging; his bowels were kept soluble. His appearance when I again saw him, which was four days after, was much improved; at the end of the first week I heard from him, and he had got to his work.

### CASE VII.

Mrs. Buckham, of a delicate constitution and slender make, begun with the fever on the morning of the 15th of June; she had frequent chills, succeeded by heat, during the day, which were very much increased towards night. When I first saw her she had severe sickness and vomiting, pain in her head, sore throat, no appetite, her face was turgid, and she was very full of the scarlet eruption; she was bled to the quantity of ten ounces, and in other respects was treated in the same way as the above; her recovery was very rapid and without any œdema.

I have also practised phlebotomy on Mr. William Gascoign, aged 17; and about a month ago on Mr. Thomas Rippon, aged 25, with the best effects; both of them had the disease in a very dangerous form.

The scarlatina anginosa has, since 1809, visited almost every little village in this neighbourhood, and frequently in a very awful form, but in no one instance, that I have seen or heard of, was its virulence so very terrible, or had spread more alarm in this part of the country than in Mr. Cumming's family; and it is also worthy of notice, that Cornsey, and Hall-hill, comparatively are two of the highest situations, from the level of the sea, of any in this neighbourhood, and had hitherto been remarked for being particularly healthy, and

* This accident, instead of retarding, seemed to accelerate the cure.

the the air pure in the extreme; and it is likewise rather extraordinary that the *miasma*, whatever it be, should have spread its baneful influence with a rapidity equal, if not greater, in those situations, than what has been generally observed in the most confined places. I should feel the highest gratification in seeing this phenomenon accounted for by an abler pen. If I may be allowed an opinion of this fact, it is that the situations of those dry and airy places are certainly less favourable to the production of the *miasma;* but by whatever means it once is either generated or has invaded such situations, the contagion will meet with less resistance, and spread its destructive qualities with greater rapidity under the influence of a light atmosphere, than where the air is more dense and loaded with grosser particles.*

* These facts in a favour of a practice deemed rash by most practitioners, will obtain, we trust, all the attention which their importance demands. They strongly indeed, confirm the mode of treatment so ably pursued, and candidly stated by Dr. Hamilton and his nephew, in a former number of this Journal; we therefore solicit further communications on this very momentous subject. If the experience of other practitioners be equally favourable, we may entertain very probable hopes that the fatality of Scarlatina Anginosa, may be in a great degree obviated. In adopting this decisive practice, however, it must not be forgotten, that the beginning of the disorder is the only period at which we might expect it would be safe, or productive of essential benefit to the patient.

